# Inhalant abuse: An overlooked problem

**DOI:** 10.4103/0019-5545.49463

**Published:** 2009

**Authors:** Sumit K. Gupta, Sonali Bali, R. C. Jiloha

**Affiliations:** Department of Psychiatry, Govind Ballabh Pant Hospital and Maulana Azad Medical College, New Delhi-110 001, India. E-mail: drsumit@aol.in

Sir,

The abuse of inhalants is an underreported and overlooked problem. The article published in last issue of the journal encouraged us to share our cases.[1]

A 14 year old schoolboy was brought to de-addiction clinic with complaints of inhaling typewriter correction fluid, lying to escape punishment, truancy from school, bullying younger brother, stealing money, running-away from home and declining academic performance. He would find time for huffing typewriter fluid after school when his parents were at work. His parents worked as sweepers and belonged to lower Socio-economic status. His father was alcohol dependent and patient had witnessed him taking alcohol and fighting with his mother in drunken state on several occasions. The locality in which they resided had many children using inhalants and some were patient's friends.

He was managed conservatively. He was psycho-educated about harmful effects of his behaviour. Parents were explained about behavioural reinforcement techniques and impact of role models on child behaviour. His father was enrolled in de-addiction clinic. For last three months, both patient and his father are maintaining abstinence and are on regular follow up. There is marked improvement in home atmosphere and bonding among family members.

In another case, Psychiatric consultation was sought for a 12 year old boy admitted in Orthopaedic Ward of associated Hospital. Patient was brought there in unconscious state after fall from a running train and both his legs and all fingers of right hand had to be amputated. On gaining consciousness on third day, patient was able to give his details.

The boy was a student of seventh standard and resided in a suburb of Delhi. Six months back, he came in contact with a neighbourhood child who used to inhale an adhesive used to fix punctured tyres. He started using the substance. He would collect money by begging outside a mosque and thrive on free food distributed by pilgrims and reside in the park facing the mosque. According to the patient, the park was residence of hundreds of persons of different age groups who abuse a variety of substances. He met with the accident after he fell out of the train in intoxicated state. His parents were illiterate and his father was an unskilled labourer. They belonged to lower socioeconomic status. Patient was managed conservatively and a rehabilitation plan was discussed with his parents.

These cases highlight that Inhalant abuse is a unique problem, as these substances are easily available, cheap and are not regulated by laws. The above cases highlight that it is more of a social problem stemming from poverty, parental neglect, poor role modelling, soft laws, changing societal norms; and thriving on child labour and beggary. The cases revealed the rampant use of such substances, although the reported cases are few; which implies that the problem is being overlooked. There is need of further research in this area.
